# Relationship between a Novel Polymorphism of the C5L2 Gene and Coronary Artery Disease

**DOI:** 10.1371/journal.pone.0020984

**Published:** 2011-06-16

**Authors:** Ying-Ying Zheng, Xiang Xie, Yi-Tong Ma, Yi-Ning Yang, Zhen-Yan Fu, Xiao-Mei Li, Xiang Ma, Bang-Dang Chen, Fen Liu

**Affiliations:** 1 Department of Cardiology, First Affiliated Hospital of Xinjiang Medical University, Urumqi, People's Republic of China; 2 Xinjiang Key Laboratory of Cardiovascular Disease Research, Urumqi, People's Republic of China; Mayo Clinic, United States of America

## Abstract

**Background:**

C5L2 has been demonstrated to be a functional receptor of acylation-stimulating protein (ASP), which is a stimulator of triglyceride synthesis or glucose transport. However, little is known about the variations in the coding region of the C5L2 gene and their association with coronary artery disease (CAD).

**Methodology/Principal Findings:**

We identified a novel single nucleotide polymorphism (SNP), 698C>T (P233L), in exon 2 using a polymerase chain reaction direct-sequencing method. This nucleotide change causes the amino-acid order from proline to leucine at codon 233. We examined the role of this SNP for CAD using two independent case–control studies: one was in the Han population (492 CAD patients and 577 control subjects) and the other was in the Uygur population (319 CAD patients and 554 control subjects). Heterozygote carriers of the 698CT genotype were more frequent among CAD patients than among controls not only in the Han population (7.3% *versus* 1.7%) but also in the Uygur population (4.7% *versus* 1.6%). The odds ratio (OR) for carriers of the 698CT genotype for CAD was 4.484 (95% confidence interval (CI): 2.197–9.174) in the Han group and 2.989 (95% CI: 1.292–6.909) in the Uygur population. After adjustment of confounding factors such as sex, age, smoking, alcohol consumption, hypertension, diabetes, as well as serum levels of triglyceride, total cholesterol, high-density lipoprotein, the difference remained significant in the Han group (*P*<0.001, OR = 6.604, 95% CI: 2.776–15.711) and in the Uygur group (*P* = 0.047, OR = 2.602, 95% CI: 1.015–6.671).

**Conclusion/Significance:**

The 698CT genotype of C5L2 may be a genetic maker of CAD in the Han and Uygur population in western China.

## Introduction

The etiology and pathogenesis of coronary artery disease (CAD) are likely to comprise a multifactorial disorder resulting from inheritance of several susceptibility genes, as well as multiple environmental determinants [1], [2]. Disorders of lipoprotein metabolism such as elevated levels of triglyceride (TG) as well as increased fasting blood glucose are considered to be important risk factors in the pathogenesis of atherosclerosis and CAD [3]–[5]. Therefore, many genes involved in the metabolism of glucose and lipid have been considered to be the candidate gene of CAD. These include the genes for: ATP-binding cassette A (ABCA1) [6], peroxisome proliferator activated receptor-gamma (PPARγ) [7], proprotein convertase subtilisin/kexin type 5 [8], and apolipoprotein E [9].

C5L2, a G protein-coupled receptor (GPCR), was demonstrated to be a functional receptor of acylation-stimulating protein (ASP) [10]–[12]. ASP is also known as C3a des-Arg, a stimulator of TG synthesis [13]–[16] or glucose transport [17], [18]. One study [19] demonstrated that ASP initiates a cascade of events that includes phosphorylation, β-arrestin translocation, and receptor internalization by binding to C5L2. Activation of C5L2 initiates a signaling pathway which includes activation and translocation of protein kinase C as well as translocation of the glucose transporter [20]–[22]. Activation of this pathway results in increased transport of glucose and esterification of fatty acids, leading to a net accumulation of TG stores in adipose tissue. Accordingly, the activity of C5L2 may influence the body's susceptibility to CAD.

Recently, Marcil et al. [23] identified a novel C5L2 variant (S323I) in a French–Canadian family with familial combined hyperlipidemia. This single nucleotide polymorphism (SNP) was associated with increased plasma TG, cholesterol, low-density lipoprotein-cholesterol (LDL-C), apolipoprotein B and ASP. However, our research team could not identify this SNP in the Han and Uygur population in China. According to the Internet website of the National Centre of Biotechnology Information (NCBI; (www.ncbi.nlm.nih.gov/SNP)), the genetic variants of the human C5L2 gene include 52 SNPs. Of these SNPs, only 4 SNPs (rs112564060, rs78669180, rs117238101, and rs112290646) yield a change in amino acids. To date, no more SNPs have been identified in the coding region of the C5L2 gene.

Accordingly, we screened for possible mutations and polymorphisms of the C5L2 gene and assessed the association between the genotypes of this gene and CAD in a Chinese Han population. We sought to replicate any associations detected in the Uygur population between the genotypes of this gene and CAD.

## Methods

### Ethical approval of the study protocol

This study was approved by the Ethics Committee of the First Affiliated Hospital of Xinjiang Medical University (Xinjiang, China). It was conducted according to the standards of the Declaration of Helsinki. Written informed consent was obtained from all participants.

### Subjects

Two patient populations (Han and Uygur) with CAD were studied independently. Four hundred and ninety-two Han patients and 319 Uygur patients diagnosed with CAD at the First Affiliated Hospital of Xinjiang Medical University from January 2006 to December 2009 were recruited. They acted as the first CAD group and second CAD group, respectively. The detailed diagnostic and selection criteria have been previously described [24]–[25]. Briefly, CAD was defined as the presence of at least one significant coronary artery stenosis of >50% luminal diameter on coronary angiography. Patients were excluded if they had congenital hypercoagulable status with proven disease-limiting life expectancy or had abused cocaine.

For each CAD patient group, we selected healthy participants matched for ethnicity, sex, and age as the controls. Control subjects were selected from the Cardiovascular Risk Survey (CRS) [26]–[27]. This consists of 14,618 subjects and is a multiple-ethnic, community-based, cross-sectional study designed to investigate the prevalence, incidence, and risk factors for cardiovascular diseases in the Han, Uygur, and Kazakh population in Xinjiang (west China) between June 2007 and March 2010. These individuals did not have: a history of CAD; electrocardiographic signs of CAD; regional wall motion abnormalities; relevant valvular abnormalities in echocardiograms [28]. Diabetes, hypertension, hyperlipidemia, smoking, and alcohol consumption were defined as previously described [24], [25], [29].

### Biochemical analysis

Serum concentrations of total cholesterol (TC), TG, glucose, high-density lipoprotein cholesterol (HDL-C), LDL-C, blood urea nitrogen (BUN), creatinine (Cr) and uric acid were measured using standard methods in the Central Laboratory of First Affiliated Hospital of Xinjiang Medical University as described previously [24], [25], [29].

### Primer design and C5L2 gene sequencing

Sequence information for use as a reference template was obtained from the Ensembl Genome Browser (Human, number ENSG00000134830). Sequencing primers were designed using Primer Premier 5.0 software. The sense primer was 5′AAGATGCCACTTCTA ACAACA3′ and the antisense primer was 5′GTTGAATGAAGGAAGGAATAA3′. Extraction of genomic DNA from peripheral blood samples has been described previously [24], [25]. The polymerase chain reaction (PCR) was undertaken with 50 ng of genomic DNA in a 20 µL reaction containing 10 µL of Power Mix (Beijing Biotech, Beijing, China), 9.5 µL of distilled water, and 0.2 mM of each forward and reverse primer. A GeneAmp 9700 thermal cycler (Applied Biosystems, Foster City, CA, USA) was used for PCR amplification. An initial denaturation step at 95°C for 5 min, 40 cycles of 95°C for 30 s, 56°C for 30 s, and 72°C for 1 min was followed by a final extension step of 72°C for 10 min. A 1615-base pair (bp) product was amplified was purified using ExoSAP-IT (Amersham Biosciences) according to manufacturer's instructions before it was used as a template for sequencing. Sequencing reactions were undertaken by BGI-Beijing (Beijing, China; http://www.genomics.cn).

### Genotyping of novel SNPs

**Figure 1:**
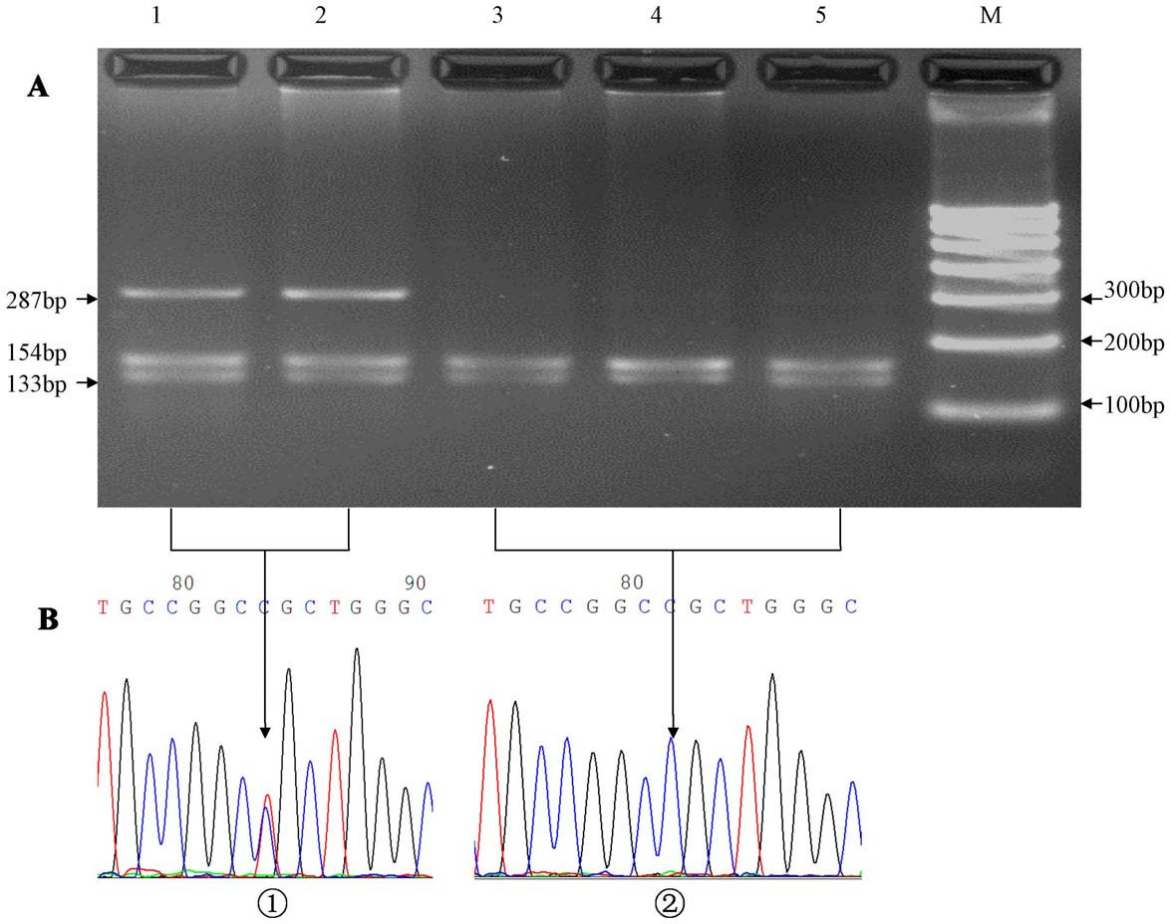
Restriction fragment length polymorphism analyses for determination of genotype (A). The CT genotype shows three bands of 286 bp, 153 bp, and 133 bp (1 and 2); the CC genotype shows two bands of 153 bp and 133 bp (3, 4 and 5). Nucleotide sequences around the 698C>T polymorphism (a, CT genotype; b, CC genotype) (**B**).

Complete sequencing of the C5L2 region that included a portion of the upstream region, 1 intron, and the exon with the complete coding region was initially conducted on 48 subjects with CAD. A novel SNP (698C>T) causes the change in amino acids from proline to leucine at codon 233. Genotyping for the 698C>T C5L2 gene variant in the present case–control study was done by PCR amplification of 286 bp in exon 2 followed by restriction digestion with *BamH* I (Fermentas, Beijing, China). The sense primer was 5′ACT ACG GCG GCT CCT CCA 3′ and the antisense primer was 5′TGT GAG CGA GGG CAA GGC 3′. The annealing temperature was 63°C. The PCR product (15 µL) was incubated overnight with *BamH* I (5 U) in a total volume of 25 µL at 37°C, and the resulting fragments separated on 3.0% agarose gel. Absence of the 698C>T variant created a *BamH* I site producing two fragments of 133 bp and 153 bp (Figure 1A). To confirm the results, we used sequenced genomic DNAs as positive controls in our assays.

### Statistical analyses

Analyses were carried out using SPSS version 17.0 (SPSS, Chicago, IL, USA). The Hardy–Weinberg equilibrium was assessed by chi-square analyses. Measurement data are shown as means ±SD, and the differences between CAD patients and control subjects were assessed by independent-sample *t*-test. Fasting TGs were log-transformed using natural logarithms for analyses and presented as geometric means and the inter-quartile range (25th–75th quartile). Differences in enumeration data between CAD patients and control subjects were analyzed using the chi-square test, as were differences in distributions of genotypes and alleles between CAD patients and control subjects. Logistic regression analyses were used to assess the contribution of the major risk factors.

## Results

### Participant characteristics

Table 1 shows the clinical characteristics of CAD patients (n = 492) and control (n = 577) Han and Uygur subjects. In Han subjects, the following variables were significantly different between the two groups: hypertension; diabetes; smoking; and the serum concentration of HDL-C, LDL-C, creatinine and BUN (all *P*<0.05). There was no significant difference in the following variables between CAD patients and control subjects: serum concentration of uric acid and TG; the body mass index (BMI); alcohol consumption; age; and sex (all *P*>0.05). In Uygur subjects, the following variables were also significantly different between these two groups: hypertension; diabetes; smoking; alcohol consumption and the serum concentration of HDL-C, TG and uric acid (all *P*<0.05). There was no significant difference in the following variables between CAD patients and control subjects: the serum concentration of LDL-C, creatinine and BUN; the BMI; age and sex (all *P*>0.05).

**Table 1:** Characteristics of the participant.

	Han				Uygur			
	Control (n = 577)	CAD (n = 492)	χ^2^ or *t*	*P* value	Control (n = 554)	CAD (n = 319)	χ^2^ or *t*	*P* value
Age, mean (SD)	57.70 (11.75)	58.45 (10.29)	1.591	0.112	49.89 (17.78)	49.69 (14.47)	−.181	0.856
Sex, female (%)	129 (0.224)	104 (0.211)	1.015	0.314	90(19.12)	62(15.38)	1.43	0.138
Hypertension, n (%)	309 (53.5)	315 (64.0)	10.979	0.001	141(26.26)	116(38.67)	13.93	<0.001
Diabetes, n (%)	167 (28.9)	241(49.0)	44.876	<0.001	63(11.87)	83(27.67)	32.91	<0.001
Smoking, n (%)	225 (39.0)	291 (59.1)	41.884	<0.001	210(38.11)	168(53.16)	17.96	<0.001
Drinking, n (%)	184 (31.9)	184 (37.4)	3.394	0.065	158(28.52)	116(36.36)	5.78	0.01
BMI, mean (SD)	25.67 (3.30)	184 (37.4)	1.563	0.118	26.37(4.04)	26.84(4.86)	−1.48	0.786
SBP, mean (SD)	133.08(18.85)	141.69(31.39)	−4.602	<0.001	141.26(29.49)	123.41(17.27)	4.777	<0.001
DBP, mean (SD)	83.74 (14.12)	86.26 (17.92)	−2.291	0.022	86.73(17.75)	76.26(10.54)	4.65	<0.001
Glucose, mean (SD)	4.88 (0.97)	6.22 (2.34)	−12.378	<0.001	5.51(1.82)	5.93(2.40)	−2.72	<0.001
TG, mean (SD)	1.85 (1.66)	2.03 (1.82)	−1.600	0.110	1.92(1.82)	1.84(0.90)	0.727	<0.001
TC, mean (SD)	4.49 (1.03)	4.17 (1.03)	4.805	<0.001	4.53(1.23)	4.21(1.04)	3.50	0.235
HDL-C, mean (SD)	1.33 (0.40)	1.13 (0.32)	8.406	<0.001	1.17(0.63)	1.00(0.28)	4.14	<0.001
LDL-C, mean (SD)	2.93 (1.02)	2.52 (0.85)	6.819	<0.001	2.68(0.94)	2.62(0.93)	0.874	0.869
UA, mean (SD)	330.64(91.68)	331.47(85.94)	−.149	0.881	304.75(87.04)	321.99(87.91)	−2.62	0.009
Cr, mean (SD)	74.25 (18.37)	78.53 (26.50)	−3.039	0.002	78.41(22.81)	78.70(21.81)	−1.71	0.120
BUN, mean (SD)	4.99(1.50)	5.29(1.72)	−3.010	0.003	5.16(1.74)	5.23(1.79)	−.521	0.602

### Distribution of the 698C>T in CAD patients and controls

The genotype distribution of this SNP did not show a significant difference from the Hardy–Weinberg equilibrium values in both ethnicities (*P*>0.05 in CAD group and control group). The frequency of the heterozygote carriers of the 698-CT genotype of *C5L2* was significantly higher in CAD patients than in Han control subjects (7.3% *versus* 1.7%; *P*<0.001) and Uygur subjects (4.7% *versus* 1.6%; *P* = 0.008) (Table 2). The frequency of the T allele in CAD patients was higher than that in Han control subjects (4.0% *versus* 1.0%, *P*<0.001) and in Uygur subjects (2.0% *versus* 1.0%; *P* = 0.008) (Table 2). The odds ratio (OR) for carriers of the 698CT genotype for CAD was 4.484 [95% confidence interval (CI): 2.197–9.174] in Han subjects and 2.989 [95% CI: 1.292–6.909] in the Uygur population. After adjustment of confounders such as hypertension, diabetes, smoking, systolic blood pressure, diastolic blood pressure, and the serum concentration of HDL-C, LDL-C, creatinine and BUN, the difference remained significant in Han subjects (*P*<0.001, OR = 6.604, 95%CI: 2.776–15.711) and in the Uygur population (*P* = 0.047, OR = 2.602, 95% CI: 1.015–6.671) (Table 3).

**Table 2:** Distribution of genotypes and alleles of C5L2 gene.

	Group	n	Genotype ( n, %)		*P*	Allele (Frequency)		*P*
			CC	CT		C	T	
Han population	Control	577	567(98.3)	10(1.7)	<0.001	0.99	0.01	<0.001
	CAD	492	456(92.7)	36(7.3)		0.96	0.04	
Uygur population	Control	554	545(98.4)	9 (1.6)	0.008	0.99	0.01	0.008
	CAD	319	304(95.3)	15(4.7)		0.98	0.02	

**Table 3:** Results of Logistic analysis.

	Uygur						Han					
	B	S.E.	Wald	P	OR	95% C.I.	B	S.E.	Wald	P	OR	95% C.I.
698C>T	0.956	0.48	3.96	0.047	2.602	1.015∼6.671	1.888	0.442	18.225	<0.001	6.604	2.776∼15.711
Smoking	0.424	0.167	6.435	0.011	1.527	1.101∼2.119	0.748	0.149	25.323	<0.001	2.112	1.579∼2.827
Diabetes	0.944	0.205	21.221	<0.001	2.57	1.720∼3.839	0.838	0.151	30.626	<0.001	2.311	1.718∼3.110
HDL-C	−1.034	0.284	13.244	<0.001	0.356	0.204∼0.621	−1.571	0.216	52.864	<0.001	0.208	0.136∼0.317
Constant	3.168	0.687	21.285	<0.001	23.771		3.168	0.687	21.285	<0.001	23.771	

### Relationship between the C5L2 genetic polymorphism and LDL-C levels

**Figure 2:**
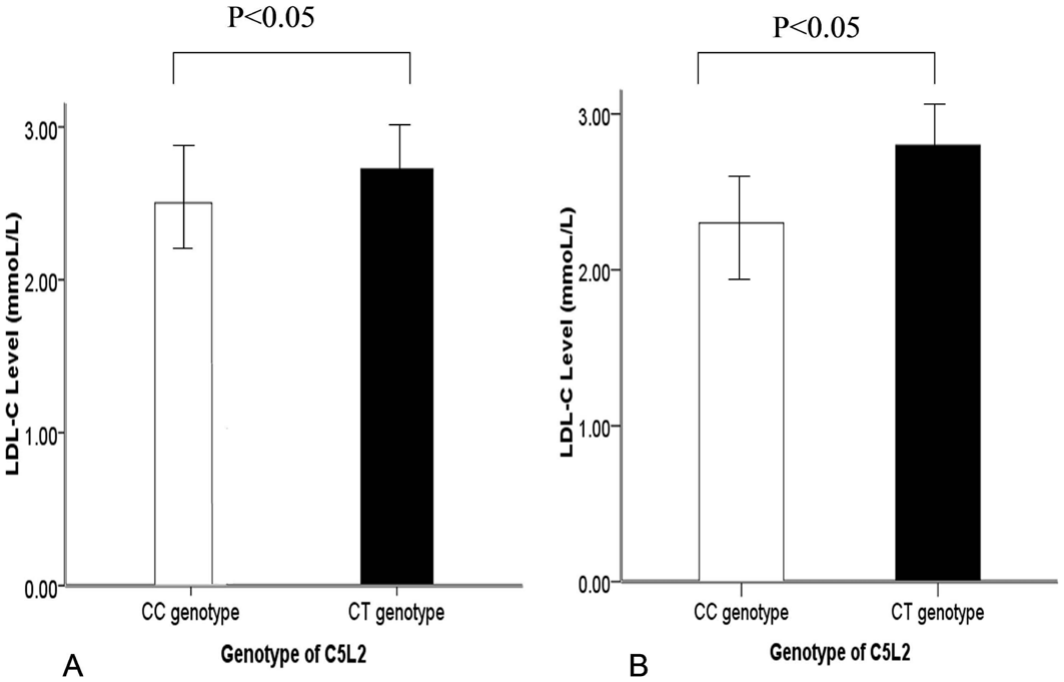
Plasma concentrations of LDL-C in patients with coronary artery disease (CAD). (**A**) In the Han population, LDL-C concentrations were significantly higher in subjects with the CT genotype than in subjects with the CC genotype. (**B**) In the Uygur population, LDL-C concentrations were significantly higher in subjects with the CT genotype than in subjects with the CC genotype.

To investigate further the functional role of the C5L2 polymorphism, we compared the concentrations of LDL-C between carriers with the CC genotype and carriers with the CT genotype of the C5L2 gene. LDL-C concentrations were significantly higher in samples from control subjects with the C5L2 CT genotype than in participants with the CC genotype in Han subjects (2.74 mmol/L *versus* 2.41 mmol/L; Figure 2A) and in Uygur subjects (2.77 mmol/L *versus* 2.36 mmol/L; Figure 2B). This results indicated that the T allelle is associated with higher LDL levels than the C allele in the control population. However the correlation between the LDL level and the T-allele is low both in CAD paitents (r = 0.11, P = 0.784) and in the control subjects (r = 0.19, P = 0.501).

## Discussion

We identified a novel SNP (698C>T) and found that the minor allele of 698C>T (SNP in C5L2) had a higher frequency in CAD patients than in controls in the Han and Uygur population of Xinjiang.

In the present study, we only selected the Han and the Uygur population acting as the participants. According to a national enquiry, 13 ethnics have been confirmed as original ethnic in Xinjiang, including the Uygur, Han, Kazak, Hui, Mongol, Kirgiz, Xibe, Manchu, Ozbek, Russian, Daur and Tatar people. Among them, the Uygur people account for 46% and the Han account for 40%. The fact that there were many ethnics in Xinjiang may be a confounding factor of the present study. So, the genetic backgrounds of these different ethnics may be helpful to understand this issue. However, Up to date, there are no completed data of these 13 ethnics' genetic backgrounds. Although there are many ethnic groups living in this area, miscegenation is very rare. When we selected the participants for the present study, we excluded those who had a history of miscegenation to ensure the homozygous nature of the samples.

Factors such as diabetes, hypertension, and hyperlipidemia have been reported to influence the pathogenesis of CAD. Like hyperlipemia and diabetes, CAD is thought to be a multifactorial disease. Hence, much attention has been focused on the association of gene polymorphisms with CAD.

The foundation for human studies examining putative causative genes that may be involved in CAD is based on a candidate gene approach. A few studies into the genetic polymorphisms of the C5L2 receptor located on chromosome 19q13 (the region identified to be associated with familial combined hyperlipidemia and the pre-diabetic state by genome-wide scan studies) have been completed [30], [31]. Familial combined hyperlipidemia is considered to be the most frequent lipoprotein disorder in premature CAD [32]. Therefore, the C5L2 gene is thought to be a candidate gene for CAD.

We genotyped the 698C>T polymorphism and assessed the association between C5L2 and CAD. The frequency of the CT genotype was significantly higher in CAD patients than in control subjects not only in the Han group but also in the Uygur group. This indicated that the risk of CAD was increased in subjects with the T allele. Logistic regression analyses suggested that, after adjustment for other cardiovascular risk factors, the CT genotype remained significant between CAD patients and control subjects.

Michel et al. [23] identified a novel variant in the C5L2 gene. This was a non-synonymous mutation; it was an AGC→ATC variant resulting in a S323-to-I substitution which was associated with familial combined hyperlipidemia in a French–Canadian family. Gain-of-function studies in human C5L2 stably transfected HEK-293 (HEK-hC5L2) cells [33] showed that TG synthesis and glucose transport were significantly increased upon ASP stimulation compared with non-transfected cells, resulting in net accumulation of adipose TG stores and insulin sensitivity. These data suggested that C5L2 was associated with hyperlipidemia and diabetes, which have been reported to be risk factors of CAD. Hence, we also examined if the relationship between the C5L2 genetic variant and CAD is modified by the concentration of TG and glucose. There was a significant difference between CAD patients and control subjects with respect to the serum concentration of glucose or the frequency of diabetes. However, it was diabetes and not the serum level of glucose that was the independent risk factor for CAD, and this relationship did not modify the association between C5L2 genetic variants and CAD.

In addition, although an elevated level of LDL-C in serum is an independent risk factor for CAD, we did not find the LDL-C level to be higher in CAD patients than in control subjects in the Han and Uygur populations. This phenomenon may be the result from the treatment of decreased-cholesterol drugs (e.g., simvastatin, lovastatin) in CAD patients. Nevertheless, to evaluate the relationship between LDL-C level and C5L2 genetic polymorphisms, as an alternative method, we compared the LDL-C level between the CC genotype and CT genotype in the control group. We found the carriers with the T allele had higher levels of LDL-C than those with the C allele.

In conclusion, in our sample of patients from west China, CAD was associated with the CT genotype of 698C>T in the human C5L2 gene. This result may broaden the knowledge of genetic variants and disease-association studies. Undertaking genome-wide association studies in different populations certainly merits investigation.

### Study limitation

The present study was limited by the relatively small sample size. This may have led to weak statistical significance and wide CIs when estimating ORs.
